# Fluid shear stress applied by orbital shaking induces MG-63 osteosarcoma cells to activate ERK in two phases through distinct signaling pathways

**DOI:** 10.1016/j.bbrep.2017.01.004

**Published:** 2017-01-11

**Authors:** Takashi Fukada, Hiroki Sakajiri, Mito Kuroda, Noriyuki Kioka, Kenji Sugimoto

**Affiliations:** a Graduate School of Life and Environmental Sciences, Osaka Prefecture University, 1-1 Gakuen-cho, Naka-ku, Sakai, Osaka 599-8531, Japan; b Graduate School of Agriculture, Kyoto University, Kitashirakawa Oiwake-cho, Sakyou-ku, Kyoto 606-8502, Japan

**Keywords:** FSS, fluid shear stress, PI3-kinase, phosphoinositide-3-kinase, Rho kinase, Rho-associated kinase, Mechanoresponse, Fluid flow, Shear stress, Osteoblast, Orbital shaking

## Abstract

Fluid shear stress (FSS) induces a series of biochemical responses in osteoblasts, and this “mechanoresponse” regulates their survival, proliferation and differentiation. However, the events in cells immediately after FSS application are unclear, and how biochemical signals from soluble factors modify the mechanoresponses is largely unknown. We used the orbital shaking method, instead of the frequently used parallel plate method, to examine activation of ERK and AKT by FSS for detailed tracking of its temporal transition. We found that ERK activation by orbital shaking was biphasic. The early phase was independent of Ca^2+^, PI3-kinase, and Rho kinase but required RAF activity. The late phase was dependent on Ca^2+^ but not RAF. These results suggest that the superior time-resolving capability of the orbital shaking method to separate the previously unrecognized Ca^2+^-independent early phase of ERK activation from the late phase. We also found that a certain combination of serum starvation and medium renewal affected ERK activation by FSS, suggesting that a soluble factor(s) may be secreted during serum starvation, which modified the phosphorylation level of ERK. These findings revealed novel aspects of the osteoblastic mechanoresponses and indicated that the orbital shaking method would be a useful, complementary alternative to the parallel plate method for certain types of study on cellular mechanoresponses.

## Highlights

• Fluid flow by orbital shaking induces biphasic activation of ERK in osteoblasts.
• Early-phase ERK activation is unique because of its independence of Ca^2+^ signaling.
• Serum starvation has complex effects on ERK activation by fluid flow.
• Orbital shaking is useful for certain types of study on cellular mechanoresponses.

## 1 Introduction

Mechanical load is an indispensable cue for proper formation, remodeling and maintenance of skeletal tissue. There are several models to explain how the hard tissue recognizes mechanical force, and an increasing number of studies support a central role of fluid shear stress (FSS) caused by interstitial fluid flow in canaliculi [1], [2], [3]. In this model, subtle distortion of the bone is converted into rapid fluid flow in canaliculi, which is detected by osteocytes and osteoblasts. However, the events in cells immediately after FSS application are unclear, and how biochemical signals from soluble factors modify the mechanoresponses is largely unknown. Information about these aspects of the mechanoresponse is important to understand the behavior of skeletal tissue under intermittent mechanical stimuli.

Currently, the parallel plate method is the most frequently used method to examine cellular responses to FSS *in vitro*. In this method, cells are sandwiched by two opposing plates, and culture medium is perfused through the narrow gap between the plates. This method creates any type of flow, such as constant or oscillatory, according to the flow pump control. Studies using this method have revealed that FSS induces Ca^2+^ signaling in cytoplasm and activation of kinases such as AKT and ERK [4], which in turn affects the expression of bone formation-related genes. However, this method has a limited throughput to follow rapid biochemical changes because of its relatively low sampling rate. It also has a disadvantage to study the interaction between autocrine/paracrine factors and mechanical stimuli, because the medium perfusion washes off and dilutes such soluble factors.

The orbital shaking method [5] is one of the alternatives to the parallel plate method. In this simple method, shaking culture dishes on an orbital shaker produces oscillatory flow of the culture medium. It can be applied to many samples simultaneously, and each sample can be easily collected at a desired time point. This feature makes the orbital shaking method suitable for following rapid cellular responses to FSS with a high time resolution. It is also suitable for studying the interaction between biochemical and mechanical signals, because the soluble factors in the medium do not wash away. However, orbital shaking cannot produce constant flow, and the magnitude and frequency of the flow oscillation are interdependent and cannot be controlled separately. Because these features of the orbital shaking method are complementary to those of the parallel plate method, each of them can be a good alternative to the other, depending on the type of assay.

We examined the activation of ERK and AKT by FSS in MG-63 cells with the orbital shaking method to closely follow its temporal transition. We found that ERK activation by orbital shaking was biphasic, and the early phase of activation was independent of Ca^2+^ signaling but required RAF activity. In contrast, the late phase was dependent on Ca^2+^ but not RAF activity. We also found that a soluble factor(s) may be secreted during serum starvation and affect ERK activation by orbital shaking. These results revealed novel aspects of the early events in mechanotransduction and showed that the orbital shaking method can be a good alternative to the parallel plate method in certain types of mechanoresponse studies.

## 2 Materials and methods

### 2.1 Materials and reagents

The MG-63 osteosarcoma cell line was provided by RIKEN BioResource Center through the National BioResource Project of the Ministry of Education, Culture, Sport, Science and Technology of Japan (Tsukuba, Ibaraki, Japan). Anti-AKT, anti-pS473 AKT, anti-p38, anti-pT180/pY182 p38, and anti-pT202/pY204 ERK1/2 antibodies were purchased from Cell Signaling Technology (Danvers, MA, USA). The anti-ERK2 antibody was obtained from Santa Cruz Biotechnology (Dallas, TX, USA). The following pharmacological inhibitors were used: BAPTA-AM (membrane-permeable calcium chelator) from Focus Biomolecules (Plymouth Meeting, PA, USA), LY294002 (PI3-kinase inhibitor) from Cayman Chemical (Ann Arbor, MI, USA), dabrafenib (RAF-1 and B-RAF inhibitor) from LKT Laboratories (Saint Paul, MN, USA), and Y-27632 (Rho kinase inhibitor) from Nacalai Tesque (Kyoto, Japan). Porcine type I collagen (Cellmatrix Type I-C) was purchased from Nitta Gelatin (Osaka, Japan). Other reagents were purchased from Nacalai Tesque unless specified otherwise.

### 2.2 Cell culture

MG-63 cells were maintained in minimum essential medium (MEM) supplemented with 10% fetal bovine serum (FBS) at 37 °C with 5% CO_2_. For orbital shaking experiments, 0.8–2×10^4^ cells were seeded in a 35-mm diameter dish that was coated with 0.3 mg/ml type I collagen in 1 mM HCl and then blocked with 0.2% bovine serum albumin (BSA) in phosphate-buffered saline (PBS). The cells were grown to ~50% confluence, washed with serum-free MEM, and then starved for serum in 1 ml serum-free MEM containing 20 mM HEPES (pH 7.2) for 24 h before FSS application.

To prepare a “peripheral only” cellular arrangement, cells in the center area (20 mm diameter) of a dish were scraped off with silicone rubber just prior to serum starvation.

For medium renewal experiments, the medium was removed from the cell culture, the cells were washed with serum-free MEM, and 1 ml fresh serum-free MEM containing 20 mM HEPES (pH 7.2) was added to the cells. To prepare mock-treated samples for medium renewal experiments, the medium was aspirated by a pipette and directly returned to the same dish.

### 2.3 Pharmacological assays

After serum starvation, a 0.2 ml aliquot of medium was collected from each culture dish in the same tube. An inhibitor in dimethyl sulfoxide (DMSO) was added to the tube at a 5× final concentration, followed by mixing, and then a 0.2 ml aliquot of this inhibitor-containing medium was dispensed to each dish and mixed by swirling. Because BAPTA-AM was not soluble in water at the 5× final concentration, its DMSO stock solution was directly added to the medium in culture dishes and then mixed by swirling. The cells were preincubated at 37 °C with inhibitors (except dabrafenib) for 1 h before FSS application. The cells were preincubated for 2 h with dabrafenib. The final concentrations of the inhibitors were: 30 µM for BAPTA-AM, and 10 µM for LY-294002, dabrafenib, and Y-27632. The final concentration of DMSO was 0.1% in all conditions.

### 2.4 FSS application and protein extraction

Dishes of serum-starved cells were placed on an orbital shaking platform in a CO_2_ incubator and shaken at 120 rpm with a 1 cm radius. This condition was estimated to produce FSS ranging from 0.33 to 2.0 Pa [6]. Each dish was collected at the indicated time, the medium was removed, and the cells were rinsed with ice-cold PBS. Lysis buffer (25 mM Tris-HCl, pH 7.5, 150 mM NaCl, 0.5% Nonidet P-40, 5 mM ethylenediaminetetraacetic acid, and 1% phosphatase and protease inhibitor mixture) was added, and the cells were scraped off. The cell suspension was transferred to centrifugation tubes and mixed in a rotary wheel for 30 min at 4 °C. Cell debris was removed by centrifugation at 15,000×*g* for 10 min, and the cleared lysate was stored at −80 °C until use. The total protein concentration in the lysate was measured by a Bradford assay.

### 2.5 Western blotting

Cell lysates containing 5 µg total protein were subjected to SDS-polyacrylamide gel electrophoresis and then transferred to a polyvinylidene fluoride membrane (Merck-Millipore, Billerica, MA, USA). Membranes were blocked with 3% BSA in TBS-T (25 mM Tris-HCl, pH 7.4, 150 mM NaCl, and 0.1% Tween-20) and then incubated with the indicated antibodies overnight at 4 °C. Signals were developed by a horseradish peroxidase-conjugated secondary antibody (GE Healthcare) and Immobilon Western Chemiluminescent HRP substrate (Merck-Millipore). Images were captured by a Luminescent Image Analyzer LAS 4000 (Fujifilm, Tokyo, Japan). Signal intensity was measured using Multi Gauge software (Fujifilm).

### 2.6 Statistical analysis

All quantitative data of the phosphorylation levels of ERK and AKT were normalized to the total protein amount of kinases before evaluations. Statistical comparison of signal intensities in a pair of treated and untreated samples was performed by Student's *t*-test using results from at least three experiments. The Dunnett contrast test was used for the multiple comparisons of the shaken/unshaken ratios of inhibitor-treated samples with the DMSO controls at each time point. A probability value of less than 0.05 was considered significant. All error bars in graphs represent the standard deviation (SD).

## 3 Results

### 3.1 Orbital shaking induces biphasic activation of ERK in MG-63 cells

**Fig. 1:**
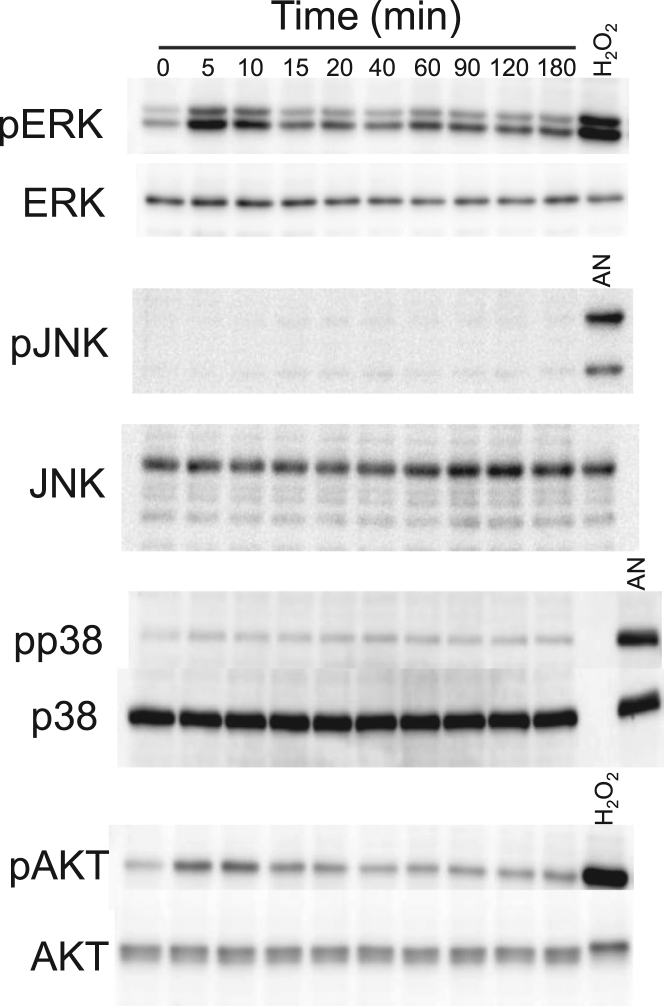
Effect of orbital shaking on the phosphorylation of ERK, JNK, p38, and AKT. MG-63 cells were exposed to orbital shaking for 5–180 min, and then phosphorylation of ERK, JNK, p38, and AKT in cell lysates was measured by western blotting. Cells treated with hydrogen peroxide (H_2_O_2_) or anisomycin (AN) were used as positive controls for kinase activation.

We applied FSS to MG-63 cells by orbital shaking and observed activation of ERK, JNK, p38, and AKT, all of which are reported to be involved in the cellular mechanoresponse. Orbital shaking induced phosphorylation of ERK and AKT, while phosphorylation of JNK and p38 was unchanged (Fig. 1). The response of ERK phosphorylation was biphasic, but AKT phosphorylation was monophasic. Because a biphasic response of ERK to FSS has not been reported, we further investigated the response of ERK and AKT to orbital shaking.

**Fig. 2:**
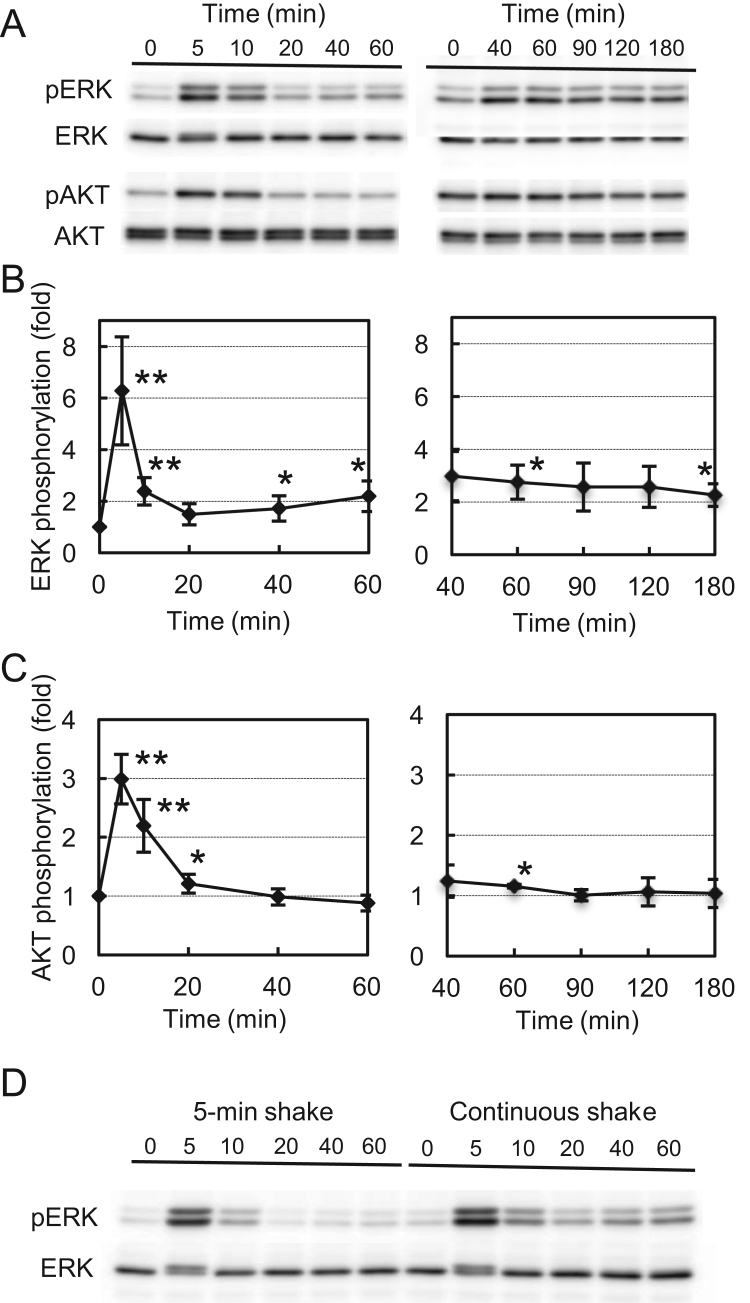
Activation of ERK and AKT induced by orbital shaking. (A) MG-63 cells were exposed to orbital shaking for 5–60 or 40–180 min, and then phosphorylation of ERK at T202/Y204 and AKT at S473 in cell lysates was measured by western blotting. (B) Signal intensities of phosphorylated ERK were normalized to total ERK and plotted as ratios to unshaken (0 min) samples in each panel of (A). (C) Signal intensities of the phosphorylated AKT in (A) were normalized to total AKT and plotted as ratios to unshaken (0 min) samples. (D) MG-63 cells were shaken for 5 min and kept static thereafter (left panel) or continually shaken for 60 min (right panel). The phosphorylation of ERK was measured by western blotting. Asterisks indicate significant difference from the basal value. Error bars, SD. *p<0.05; **p<0.01.

FSS applied by orbital shaking to MG-63 cells induced ERK phosphorylation by ~6-fold in 5 min, but this activation was transient and ceased within 20 min. ERK phosphorylation increased again by ~2-fold at 40–60 min after the onset of shaking, and this weak activation persisted for at least an hour thereafter (Fig. 2A, B). AKT phosphorylation also increased by ~3-fold in 5 min but returned to the basal level within 40 min, and remained at this level thereafter (Fig. 2A, C). When the cells were shaken for 5 min and kept static thereafter, the late response of ERK phosphorylation did not appear, indicating that continuous FSS was necessary to induce the late response (Fig. 2D).

**Fig. 3:**
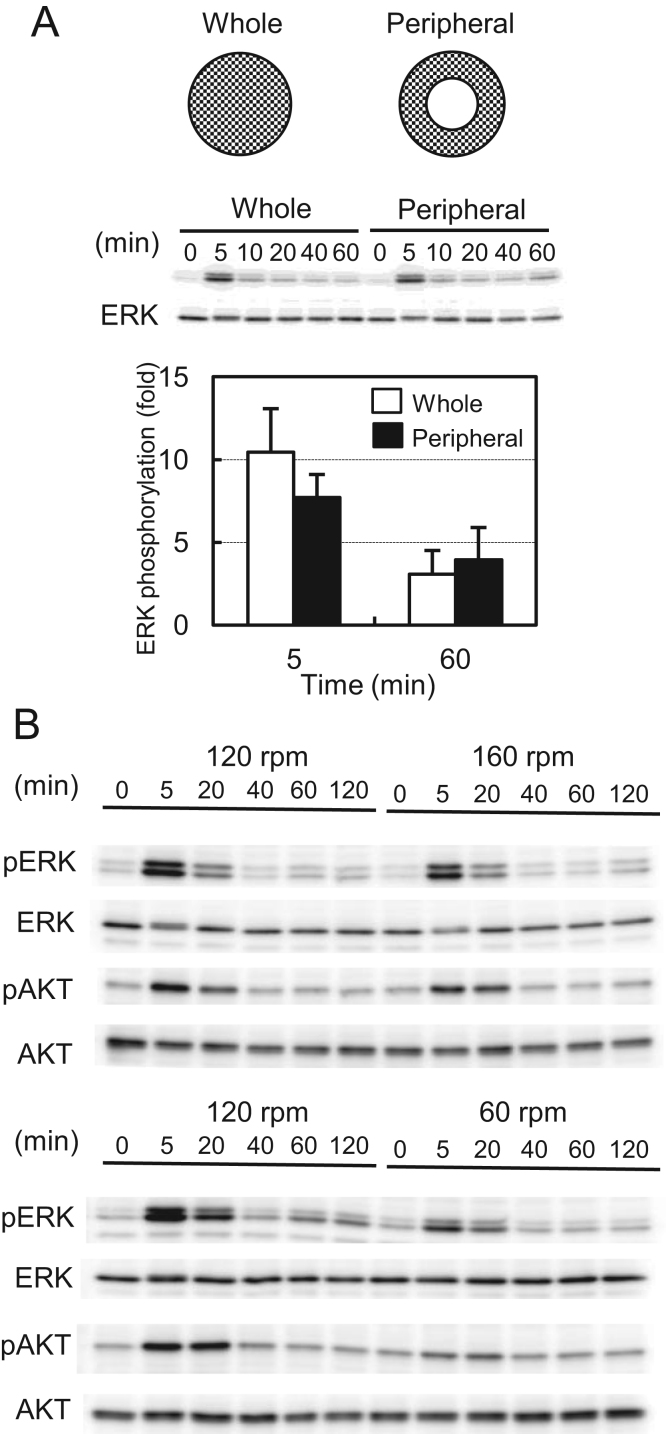
Effect of positional variation of FSS strength on ERK and AKT phosphorylation by orbital shaking. (A, upper panel) Schematic diagrams of “Whole” and “Peripheral” arrangements of the cells. (A, middle panel) MG-63 cells in whole or peripheral arrangements were exposed to orbital shaking, and then phosphorylation of ERK was measured by western blotting. (A, lower panel) Signal intensities of phosphorylated ERK were normalized to total ERK and plotted as ratios to unshaken (0 min) samples. Open bars, whole; closed bars, peripheral. Error bars, SD. *p<0.05; **p<0.01. (B) MG-63 cells were shaken at 120 and 160 rpm (upper panel) or 120 and 60 rpm for the indicated times, and then phosphorylation of ERK and AKT was measured by western blotting.

In typical studies using parallel plate assays, FSS weakly (~2-fold) activates ERK within 5 min, and the same level of activation is maintained for the duration of the fluid flow (for example, [4]). This previous study did not report the biphasic manner of ERK activation by FSS. Because FSS generated by orbital shaking is reported to steeply increase near the rim of a dish under certain conditions [7], we considered that the biphasic manner might result from the difference in FSS magnitude between the center and peripheral regions of the culture dish. To test this possibility, we removed the cells in the center area (30% of the total area) of the dishes at 24 h before shaking. This peripheral arrangement of cells did not significantly affect the biphasic manner or peak intensity of ERK phosphorylation induced by orbital shaking (Fig. 3A). Furthermore, increasing the rotational speed from 120 to 160 rpm did not enhance ERK or AKT responses. However, decreasing the rotational speed to 60 rpm weakened the overall, either early or late, responses of ERK and AKT (Fig. 3B), suggesting that the response to FSS was saturated in most cells at 120 rpm. These results indicated that the positional variation of the FSS magnitude in a culture dish was unlikely to be the cause of the biphasic activation of ERK by orbital shaking.

### 3.2 Early and late responses of ERK to FSS result from different signal transduction pathways

**Fig. 4:**
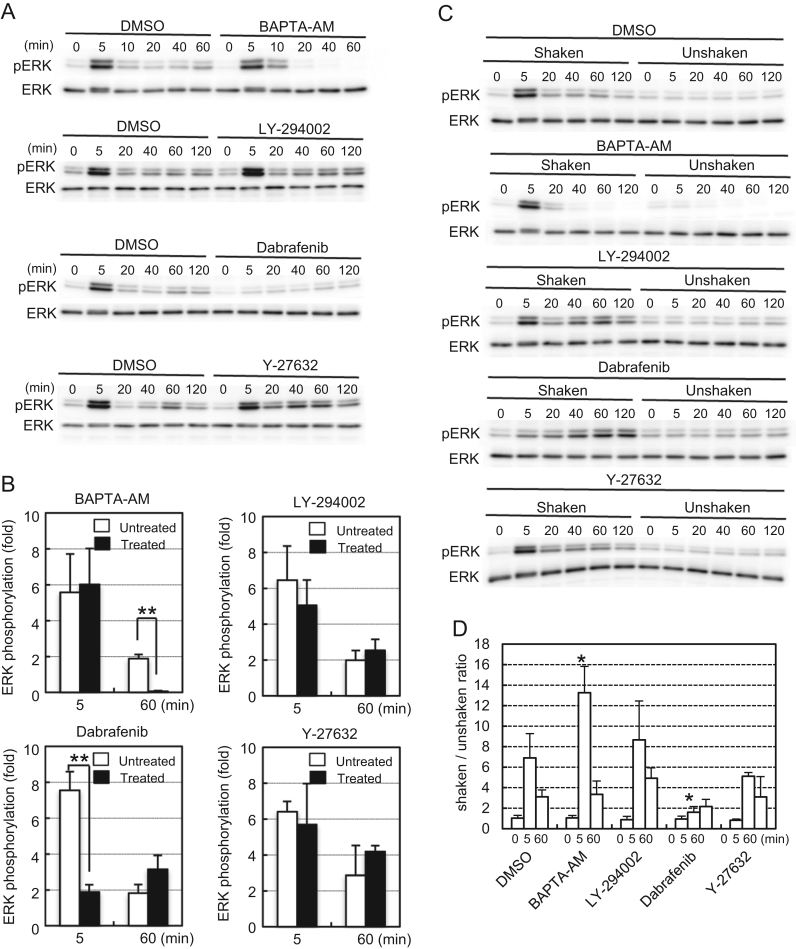
Pharmacological analysis of ERK activation induced by orbital shaking. (A) MG-63 cells were exposed to orbital shaking for the indicated times in the presence or absence of an inhibitor, and then phosphorylation of ERK was measured by western blotting. (B) Signal intensities of the phosphorylated ERK in (A) were normalized to total ERK and plotted as ratios to unshaken (0 min) samples. Open bars, DMSO controls; closed bars, inhibitor-treated samples. (C) MG-63 cells were exposed to orbital shaking or kept static for the indicated times in the presence of the same inhibitor, and then phosphorylation of ERK was measured by western blotting. (D) Signal intensities of phosphorylated ERK in (C) were normalized to total ERK and plotted as shaken/unshaken ratios. Error bars, SD. *p<0.05; **p<0.01.

**Fig. 5:**
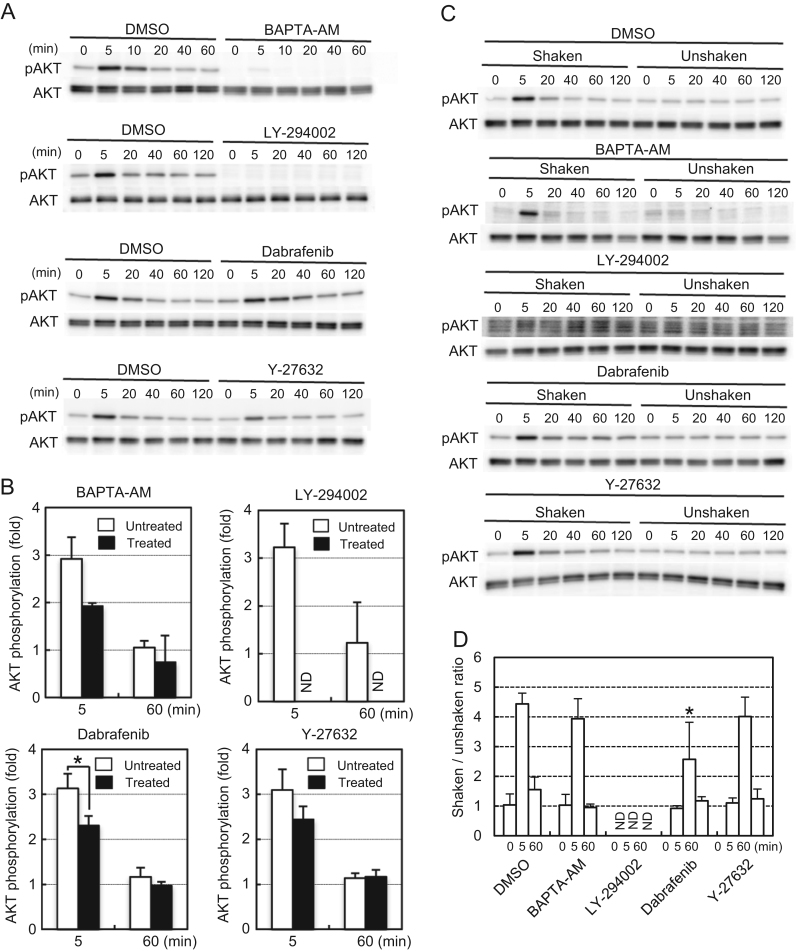
Pharmacological analysis of AKT activation induced by orbital shaking. (A) MG-63 cells were exposed to orbital shaking for the indicated times in the presence or absence of an inhibitor, and then phosphorylation of AKT was measured by western blotting. (B) Signal intensities of phosphorylated AKT in (A) were normalized to total AKT and plotted as ratios to unshaken (0 min) samples. Open bars, DMSO controls; closed bars, inhibitor-treated samples. (C) MG-63 cells were exposed to orbital shaking or kept static for the indicated times in the presence of the same inhibitor, and then phosphorylation of AKT was measured by western blotting. (D) Signal intensities of phosphorylated AKT in (C) were normalized to total AKT and plotted as shaken/unshaken ratios. Error bars, SD.* *p*<0.05; **p<0.01. ND, not detected.

We next examined whether the early and late responses of ERK to FSS were induced by different biochemical signaling pathways by pharmacological analyses. The responses of ERK (Fig. 4) and AKT (Fig. 5) were analyzed by two indexes. First, we compared the kinase phosphorylation levels under FSS in the presence or absence of an inhibitor (Figs. 4A and 5A). The relative phosphorylation ratio was calculated by setting the signal intensity at 0 min of inhibitor-treated and untreated samples, respectively, as 1 (Figs. 4B and 5B). The second index was the shaken/unshaken ratio in the presence of an inhibitor (or DMSO as a control). We compared the phosphorylation levels of the kinases with or without FSS at each time point in the presence of the same inhibitor (Figs. 4C and 5C), and calculated the shaken/unshaken ratio as an index of the responsiveness of the kinase phosphorylation to FSS (Figs. 4D and 5D).

A remarkable result was that the early response of ERK to FSS did not depend on cytoplasmic Ca^2+^. BAPTA-AM, a membrane-permeable calcium chelator, did not affect the early phosphorylation of ERK (Fig. 4A, B), and it enhanced the responsiveness of the early ERK phosphorylation to FSS (Fig. 4C, D). The late phosphorylation of ERK appeared to be responsive to FSS in the presence of BAPTA-AM (Fig. 4D), but the amount of phosphorylated ERK protein was negligible in comparison with the DMSO control (Fig. 4A, B). These results raised the possibility that, unlike the late response, a novel Ca^2+^-independent mechanism induced the early response of ERK. Therefore, we characterized the involved signaling pathway by further pharmacological analyses.

Inhibition of PI3-kinase or Rho kinase by LY-294002 or Y-27632, respectively, did not affect ERK activation. Dabrafenib, an inhibitor of RAF-1 and B-RAF, suppressed the early activation of ERK, but a significant amount of phosphorylated ERK remained after 60 min of shaking in the presence of dabrafenib (Fig. 4A), which gradually increased over time only when FSS was applied (Fig. 4C). These results suggested that FSS induced the early phase of ERK activation *via* the RAF/MEK/ERK pathway, whereas the late phase was induced in a Ca^2+^-dependent and RAF-independent manner.

AKT was responsive to FSS in the presence of BAPTA-AM (Fig. 5C, D), but its basal and induced phosphorylation levels were much lower than those in the DMSO control (Fig. 5A), indicating that Ca^2+^ was required to maintain the substantial proportion of AKT phosphorylation. LY-294002 abolished the basal and induced phosphorylation of AKT, and dabrafenib slightly reduced the FSS-induced phosphorylation of AKT. Inhibition of Rho kinase by Y-27632 did not affect the FSS-induced AKT phosphorylation. These results suggest that the FSS-induced AKT activation is primarily dependent on PI3-kinase, and requires Ca^2+^ signaling and RAF activity for full activation.

### 3.3 A certain combination of serum starvation and medium renewal increases basal phosphorylation of ERK and reduces the relative magnitude of its early phase of activation

**Fig. 6:**
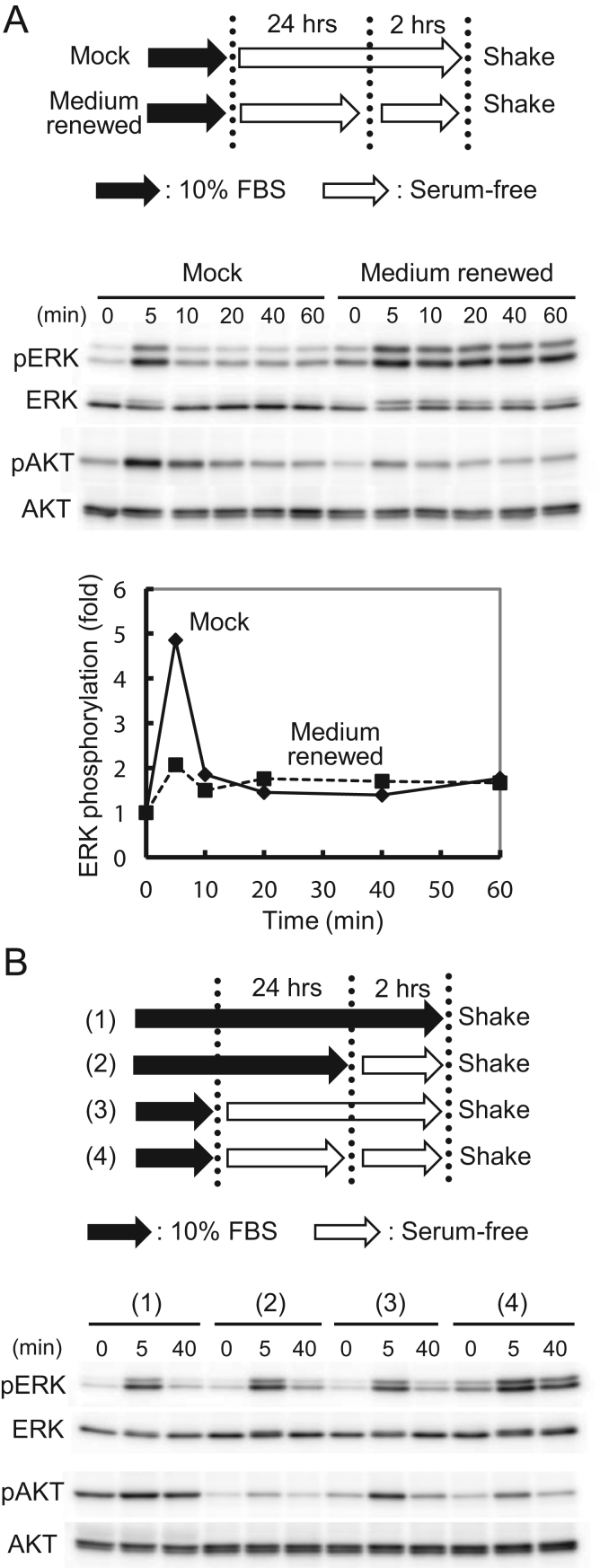
Combinational effect of serum starvation and medium renewal on the activation of ERK by orbital shaking. (A, upper panel) Schematic diagram of the serum starvation and medium renewal schedules. (A, middle panel) Western blots showing ERK and AKT activation by orbital shaking with or without medium renewal. (A, lower panel) Signal intensities of phosphorylated ERK in (A) were normalized to total ERK and plotted as ratios to unshaken (0 min) samples. (B, upper panel) Schematic diagram of the serum starvation and medium renewal combinations. Closed arrows: incubation in medium containing 10% FBS; open arrows: incubation in serum-free medium. (B, lower panel) Western blots showing ERK and AKT activation by orbital shaking with combinations of serum starvation and medium renewal.

In bone canaliculi, soluble factors secreted from cells can remain in the narrow extracellular space for long time and affect the behavior of neighboring cells. An orbital shaking system has a potential advantage over parallel plate systems to study the effects of such soluble factors on the cellular mechanoresponse because perfusion of the culture medium in a parallel plate system quickly washes away soluble factors secreted from the cells. To analyze the effects of such secreted factors, we replaced the medium of the serum-starved cells with fresh serum-free medium at 2 h before beginning the orbital shaking to characterize the potential effect of soluble factors that may be secreted during the serum starvation in our experimental settings. As shown in Fig. 6A, this treatment increased the basal and late phosphorylation levels of ERK, while little effect was found on the early peak of phosphorylation. As a result, the activation ratio of ERK to the basal value was diminished at the early peak (Fig. 6A, lower panel). We observed this increase in basal phosphorylation only when the cells were subjected to both 24 h of serum starvation and medium renewal. Either of the treatments alone showed no effect on ERK activation by FSS (Fig. 6B). The response of AKT phosphorylation to these treatments was different from that of ERK, suggesting this combinational effect of serum starvation and medium renewal is specific to ERK.

## 4 Discussion

In this study, we examined ERK and AKT activation by orbital shaking to determine the time course of their responses to FSS in detail. The orbital shaking method is suitable for following rapid responses to FSS owing to its high time-resolving capability. Using this method, we found that ERK activation by FSS was biphasic, consisting of a strong, transient activation (early response) and subsequent weak, persistent activation (late response). Although Liu et al. have reported that strain force produced by four-point bending induces similar biphasic activation of ERK [8], we could not find reports describing a biphasic ERK response to FSS. ERK shows a biphasic response when activated by growth factors such as epidermal growth factor, and this biphasic manner is thought to be the result of negative feedback loops in the RAS/RAF/MEK/ERK pathway [9]. However, because we found that the early and late activations of ERK by FSS were different in terms of their dependence on cytoplasmic Ca^2+^ and RAF activity, the biphasic response of ERK to FSS is unlikely to be the result of negative feedback.

Most previous studies on ERK responses to FSS have employed parallel plate assays and reported 2–3-fold activation of ERK, but there is a limited amount of data on ERK activation within 5 min of FSS application. Lee et al. reported that oscillatory fluid flow in a parallel plate chamber induces constant ~2-fold activation of ERK in MG-63 cells at 5 min–24 h after the onset of flow [4]. Young et al. used a parallel plate device and orbital shaker to apply oscillatory flow to MG-63 cells, and reported that ERK phosphorylation increased by 2–3-fold after 5–15-min exposure to the flow in either system [10]. However, because Young et al. provided data only up to 30 min, their report did not show whether secondary activation followed the first. In general, the magnitude, time course, and Ca^2+^-dependence of ERK activation shown in previous reports suggested that, in most cases, the reported ERK response to FSS probably corresponds to the late response we observed in this study, and the high time-resolving capability of the orbital shaking method separated the previously unrecognized Ca^2+^-independent early phase of ERK activation from the late phase. However, we cannot rule out the possibility that the different types of flow caused the different manners of ERK activation because the fluid flow in a dish under orbital shaking is more complex and dynamic than the laminar flow in a parallel plate chamber [7].

**Fig. 7:**
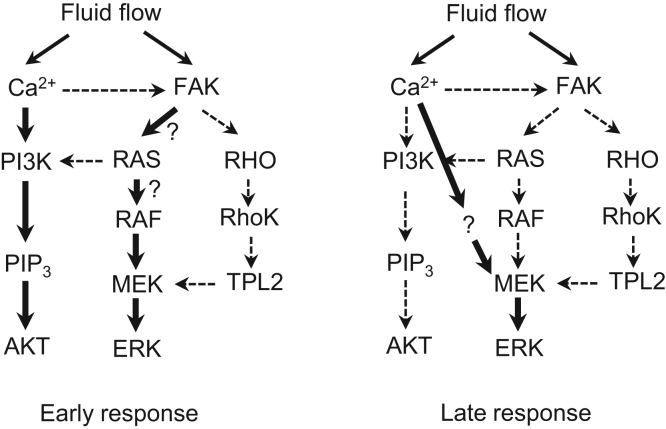
Schematic diagrams of the pathway models involved in the early and late phases of ERK and AKT activation by orbital shaking. Arrows indicate some of the known pathways associated with ERK and AKT activation by FSS. Thick arrows indicate the pathways that are likely involved in the activation of ERK and AKT by orbital shaking in this report. Dotted arrows indicate the pathways that are unlikely to contribute to the ERK and AKT activation by orbital shaking.

We found that the early activation of ERK by orbital shaking was independent of Ca^2+^, PI3-kinase, and Rho kinase (Fig. 4), although most of the reported signaling pathways involved in mechanoresponses are dependent on some of these transducers [2], [4], [11], [12], [13]. We did not identify the transducers acting upstream of RAF in the early activation of ERK, although the FAK/SOS/RAS pathway would be a good candidate (Fig. 7). A previous study has reported that induction of osteopontin and cyclooxygenase 2 gene expression by FSS is independent of Ca^2+^ signaling, but dependent on primary cilia in MC3T3-E1 and MLO-Y4 osteocyte-like cells [14]. The relationship of primary cilia and the Ca^2+^-independent ERK activation we observed in MG-63 cells remains to be elucidated.

We found that inhibition of PI3-kinase did not affect the ERK activation by FSS (Fig. 4). This finding is inconsistent with a reported result in which a PI3-kinase inhibitor suppressed ERK activation by FSS, even though they also used MG-63 cells [4]. Their report also showed that the PI3-kinase inhibitor blocked induction of ERK and AKT phosphorylation by FSS without affecting their basal phosphorylation. In this study, we found that the PI3-kinase inhibitor suppressed both the induced and basal phosphorylation of AKT (Fig. 5). The reason for these discrepancies is not apparent, but there are some differences in experimental conditions other than the type of FSS application. They used fibronectin-coated glass plates for cell culture, whereas we used collagen-coated dishes. They starved the cells for serum in medium including 0.5% FBS, but we used serum-free medium. These differences appear subtle but may have complex effects on ERK and AKT phosphorylation.

Our results also showed the importance of observing kinase phosphorylation by two indexes in inhibitor experiments: the relative phosphorylation ratio to the basal value and the shaken/unshaken ratio. The relative phosphorylation ratio to the basal value was suitable to observe the effect of inhibitors on the overall phosphorylation levels of the kinases. However, in some cases, preincubation of the cells with an inhibitor changed the basal phosphorylation level at 0 min, which markedly affected the relative phosphorylation ratio. In contrast, the shaken/unshaken ratio was not affected by the variation of the basal phosphorylation level. However, in some cases, the peak phosphorylation level was negligible even when the shaken/unshaken ratio was fully responsive to FSS. Both indexes should be measured to observe the actual behavior of cells in the inhibitor experiments.

We found that basal and late phosphorylation of ERK was increased by a certain combination of serum starvation and medium renewal before the application of FSS (Fig. 6). This effect diminished the activation ratio of ERK, especially at the early peak of phosphorylation, and may partly explain why the biphasic manner of the FSS response was not apparent in parallel plate assays. The effect of serum starvation and medium renewal on AKT phosphorylation (Fig. 6) appears to be easy to explain. The high phosphorylation level of AKT was supported by growth factors in serum, but this support ceased after 2 h of serum starvation. AKT phosphorylation partly recovered after 24 h of starvation, probably by accumulation of autocrine factors, but their effects were lost by medium renewal. However, this scheme does not appear to be the case for ERK phosphorylation. Because the medium renewal mainly affected the basal ERK phosphorylation, this effect is likely to be a part of the general regulation of ERK activity, which consequently affects the ERK response to FSS, rather than a mechanoresponse-specific effect. It is possible that the combination of serum starvation and medium renewal induced a stress response that affected the basal phosphorylation of ERK. However, more investigation is necessary to clarify this point.

In summary, we found that the FSS created by orbital shaking induced ERK activation in a biphasic manner, and the early phase was independent of Ca^2+^ signaling but dependent on RAF activity. Because there are few reports describing Ca^2+^-independent activation of ERK by FSS, our findings suggest that a previously unrecognized transduction pathway is involved in the early events of the mechanoresponse. Considering that most mechanical stimuli *in vivo* are intermittent, analysis of the early phase of the mechanoresponse would be important.

Orbital shaking is not currently a major method to measure the cellular response to FSS, but it has features that are complementary to those of the parallel plate method. These features of the orbital shaking method provide the basis for our findings in this study and showed that each of the two methods would be a good alternative to the other, depending on the type of study.
